# Primary or secondary prevention of HIV-associated histoplasmosis during the early antiretrovirals for all era

**DOI:** 10.1371/journal.pntd.0011066

**Published:** 2023-02-02

**Authors:** Mathieu Nacher, Paul Le Turnier, Philippe Abboud, Ugo Françoise, Aude Lucarelli, Magalie Demar, Félix Djossou, Loïc Epelboin, Pierre Couppié, Antoine Adenis

**Affiliations:** 1 CIC INSERM 1424, Centre Hospitalier de Cayenne, Cayenne, French Guiana; 2 Département Formation Recherche, Université de Guyane, Cayenne, French Guiana; 3 Service des maladies infectieuses et tropicales, Centre Hospitalier de Cayenne, Cayenne, French Guiana; 4 Coordination Régionale de la lutte contre le VIH et les infections sexuellement transmissibles Guyane, Centre Hospitalier de Cayenne, Cayenne, French Guiana; 5 Unité mixte de recherche Tropical Biome and Immunopathology, Université de Guyane, Cayenne, French Guiana; 6 Laboratoire de Parasitologie-Mycologie, Centre Hospitalier de Cayenne, Cayenne, French Guiana; 7 Service de Dermatologie, Centre Hospitalier de Cayenne, Cayenne, French Guiana; Albert Einstein College of Medicine, UNITED STATES

## Thinking anew about prophylaxis in the “treat all” era

Although most studies actually concern symptomatic patients, the natural history of HIV-associated progressive disseminated histoplasmosis is presumably often gradual; as the immunity gradually declines, the fungal burden gradually increases, and the intensity of symptoms, especially those impairing vital functions, grows. Hence, in this view, early on, patients have few or no symptoms until the fungal burden reaches symptomatic levels. Presently, much of the attention still focuses on diagnosing symptomatic patients because it remains such a widespread problem. Intuitively, the answer whether primary or secondary prevention is useful would be a resounding yes. But since 2017, it is recommended that all persons living with HIV should be on highly active antiretroviral therapy (HAART), which is a major change that may also affect the elements weighted in the decision to start primary or secondary prevention against histoplasmosis. Recently, primary prophylaxis of *Pneumocystis* pneumonia, perhaps the most emblematic prophylaxis in HIV care, was reevaluated. Arguing that “adding prophylactic treatment, apart from increasing pill burden, could cause adverse events and potentially increase the risk of antibacterial resistance due to prolonged usage,” using an emulated trial, the COHERE collaboration have suggested that primary prophylaxis might be safely withheld in confirmed virologically suppressed patients on ART, regardless of their CD4 count [1,2].

In the present article, we try to answer the question on whether primary or secondary prevention of disseminated histoplasmosis could still be useful in the early antiretrovirals for all era.

## Itraconazole prophylaxis

The Infectious Diseases Society of America has recommended primary prophylaxis for persons with CD4 counts <150/mm3 when the annual incidence of histoplasmosis exceeded 10 per 100 person-years [3]. To our knowledge, the main study on itraconazole prophylaxis was a prospective randomized double-blind placebo-controlled study in patients with CD4 <150/μL studying the efficacy and tolerance of itraconazole (200 mg/day) in endemic areas in the United States of America [4]. In patients with CD4 counts <100/μL, itraconazole significantly reduced cases of histoplasmosis 7/103 (7%) for placebo recipients versus 2/101 (2.0%) in those receiving itraconazole. It was generally well tolerated but there were significantly more discontinuations for side effects in the itraconazole group than in the placebo recipients, 8.7% versus 3.4%, respectively. Adherence was insufficient with nearly 40% of patients no longer taking the allocated treatment and itraconazole dosage being undetectable also in 40% of patients. Furthermore, the proportion of *Candida* isolates recovered from subjects on itraconazole that were susceptible to itraconazole was significantly lower than in those receiving placebo (63% versus 96%, respectively) [5]. Although, it is summarily reported that itraconazole prophylaxis prevented histoplasmosis but did not improve survival, there are a number of circumstances that suggest that larger studies may have found otherwise. First, it was a small study and the incidence of histoplasmosis was lower than initially expected. Furthermore, with nearly 40% of patients not on their allocated treatment at the end of the study, the per protocol analysis showed that among the 4 cases of histoplasmosis in the itraconazole arm, in fact, 3 out of the 4 were no longer taking itraconazole. Only 1 death was linked to histoplasmosis (it was in the itraconazole group). In these circumstances, it seems impossible to conclude anything about the outcome of death from histoplasmosis: Absence of evidence was more a result of low statistical power than evidence of absence of effect. Although the study was preHAART, it showed that patients who were being treated with 3 or more antiretroviral drugs at the completion of the study were significantly less likely to have prophylaxis failure.

## Highly active antiretroviral therapy (HAART)

HAART has since shown a potent effect on reducing the incidence of disseminated histoplasmosis [6]. Hence, after adjusting for age, sex, and CD4 counts, HAART was associated with an 80% reduction of histoplasmosis incidence (adjusted HR = 0.2 (0.1 to 0.4), *P* < 0.001). Furthermore, in those with histoplasmosis, HAART was also associated with a significant reduction of death at 6 months (adjusted HR = 0.2 (0.0 to 0.5), *P* = 0.003) after adjusting for age, sex, CD4, and CD8 counts [6].

## Evidence for de novo infections and asymptomatic/paucisymptomatic infections in endemic areas

In a cohort of HIV-infected persons living in endemic French Guiana, where the prevalence of positive skin tests is around 30%, we found that the incidence rate of disseminated histoplasmosis increased sharply as CD4 counts dropped below 100 per μL exceeding 10 per 100 person years, with a significant seasonal effect and a risk difference of 0.7 per 100 person years between dry and wet seasons [7,8]; the attributable risk percent in the overall HIV cohort (61%) implies that, overall, 61% of cases were attributable to the dry season and, arguably, to newly acquired infections. Hence, for patients in our HIV cohort, and presumably most cohorts in endemic Latin America [9], there is regular exposure to a major pathogen for most patients and thus possibility of prevention of infection and dissemination.

Evidence for a spike of incidence shortly after initiating HAART [7] suggests that immune reconstitution had revealed previously undetected latent histoplasmosis in these patients. In a prospective study between 2016 and 2018 in Cayenne hospital, 158 patients with CD4 <200 were tested using the IMMY Clarus EIA. Overall, 14/87 symptomatic patients had positive antigen detection (16.1% [8.4% to 23.8%]) and 2/71 asymptomatic patients had positive antigenemia (2.8% [0.3% to 9.8%]).

## Before the diagnosis: Then and now

In a cohort of 343 patients (see [10] for details of the cohort) with confirmed diagnosis of first episode of disseminated histoplasmosis for whom data was available for the year of HIV infection and the year of histoplasmosis, we found that the median delay between HIV infection and histoplasmosis was 2 years (IQR = 0 to 7 years) (S1 Fig). However, over time, with most patients receiving HAART, since 2010, the delays and the number of cases have dropped (S2 Fig). Nevertheless, since the “treat all” strategy in 2016, there were 17 histoplasmosis cases, 5/17 were patients on HAART and 3/5 developed histoplasmosis over a year after their HIV diagnosis. None of these 3 patients was virologically suppressed. Although poor adherence was probable, this suggests that, had itraconazole been prescribed, this could have been prevented.

For a subset of 243 patients with disseminated histoplasmosis, information was available on the interval between initial symptoms and hospitalization; in the past decade, the median interval ranged between 10 and 30 days (S3 Fig).

Overall, this shows that, despite a universal health system, despite intense HIV testing (203 tests per 1,000 inhabitants per year), delays for HIV diagnosis are widespread [11] and that patients are often hospitalized after substantial delays for a disease, which, when severe, may lead to deaths in a matter of days.

## Primary or secondary prevention?

We have seen that many patients acquire histoplasmosis in endemic areas, that some patients with histoplasmosis are asymptomatic, that itraconazole prophylaxis is effective to reduce incidence, that it seems cost effective [12]; we have also seen the powerful impact of HAART on the incidence of symptomatic histoplasmosis. Since a substantial proportion of cases of histoplasmosis are diagnosed the same year as the HIV diagnosis, which entails rapid HAART initiation, one could question whether itraconazole primary prophylaxis still could add benefits since ARVs often allow a rapid increase in CD4 counts. However, it is arguable that the actual incidence rate of disseminated histoplasmosis in patients with less than 100 CD4/μL will remain in a dangerous zone until CD4 increase is sufficient. Indeed, it has been shown that CD4 recovery is much slower among those with initial CD4 counts below 100 per μL, precisely those who have the highest risk of disseminated histoplasmosis, and that a year after initiating HAART, a substantial fraction may still have CD4 counts in the dangerous zone [13,14]. Despite adoption of “Treat All” by nearly all countries in Latin America, late ARV treatment initiation is pervasive with much spatial heterogeneity and up to half of HIV patients arriving at the advanced stage, thus representing prime circumstances for histoplasmosis until HAART has restored sufficient cellular immune responses [15,16].

WHO has recently included histoplasmosis as a high-priority pathogen [17]. Patients with advanced HIV should ideally benefit from screening for histoplasmosis, as recommended by the PAHO/WHO guidelines. But in the current situation, this is still not possible in most Latin American countries and perhaps may take years to percolate. The IDSA recommended itraconazole prophylaxis when annual incidence is greater than 10%. We emphasize that this is exactly the case for persons with less than 100 CD4 counts living in much of Latin America; thus, despite the potential trade-offs between side effects and adherence, itraconazole prophylaxis may be beneficial and cost effective for this specific population, to prevent infection from *H*. *capsulatum* and to prevent dissemination of latent infections, at least until screening is rolled out in all HIV care settings. Given the lack of hard evidence, the potential trade-offs, the uncertainties about when screening will really be available, and spatial heterogeneities in the actual access to itraconazole and HAART, this important question deserves rigorous consideration by a panel of experts using the Grade methodology to issue clear guidelines for clinicians [18].

**Key Learning Points**

➢ Rapid initiation of highly active antiretroviral therapy (HAART) for all patients diagnosed with HIV rapidly reduces the level of immunosuppression.
➢ In this context, the duration of primary prophylaxis and the trade-offs between primary prophylaxis and lack of side effects for optimal HAART adherence have changed.
➢ Screening and treatment among persons with advanced HIV using *Histoplasma* antigen detection tests is still not possible in most countries given the slow rollout of diagnostic tests.
➢ Given the high incidence of disseminated histoplasmosis in the profoundly immunosuppressed, the question of primary prophylaxis against histoplasmosis for patients with advanced HIV in endemic areas should be reconsidered until *Histoplasma* antigen tests become widely available.
➢ A systematic review of the pros and cons of such a policy in the context of early antiretroviral therapy for all patients should lead to clear guidelines for clinicians in different contexts.

**Top Five Papers**

1. WHO fungal priority pathogens list to guide research, development and public health action n.d. Available from: https://www.who.int/publications-detail-redirect/9789240060241 (accessed December 30, 2022).
2. McKinsey DS, Wheat LJ, Cloud GA, Pierce M, Black JR, Bamberger DM, et al. Itraconazole prophylaxis for fungal infections in patients with advanced human immunodeficiency virus infection: randomized, placebo-controlled, double-blind study. National Institute of Allergy and Infectious Diseases Mycoses Study Group. Clin Infect Dis. 1999;28:1049–56. https://doi.org/10.1086/514744
3. Atkinson A, Zwahlen M, Barger D, d’Arminio Monforte A, De Wit S, Ghosn J, et al. Withholding primary PcP prophylaxis in virologically suppressed HIV patients: an emulation of a pragmatic trial in COHERE. Clin Infect Dis. 2020.
4. Nacher M, Adenis A, Blanchet D, Vantilcke V, Demar M, Basurko C, et al. Risk factors for disseminated histoplasmosis in a cohort of HIV-infected patients in French Guiana. PLoS Negl Trop Dis. 2014;8:e2638. https://doi.org/10.1371/journal.pntd.0002638
5. Roul H, Mary-Krause M, Ghosn J, Delaugerre C, Pialoux G, Cuzin L, et al. CD4+ cell count recovery after combined antiretroviral therapy in the modern combined antiretroviral therapy era. Aids. 2018;32:2605–14.

## Supporting information

S1 Fig Shows that the greatest proportion of patients with disseminated histoplasmosis were recently diagnosed with HIV. (TIF)

S2 Fig Shows that in the past decade, the yearly number patients with disseminated histoplasmosis declined and that the delay between HIV diagnosis and histoplasmosis diagnosis also declined. (TIF)

S3 Fig Shows that in the past decade, the delay between the first symptoms leading to the histoplasmosis diagnosis declined and remained stable oscillating between 10 and 30 days. (TIF)
