# Supplementary figures and images for: Primary or secondary prevention of HIV-associated histoplasmosis during the early antiretrovirals for all era
Source: PLoS Negl Trop Dis. 2023 Feb 2;17(2):e0011066. doi: 10.1371/journal.pntd.0011066 (PMC9894381; doi:10.1371/journal.pntd.0011066)

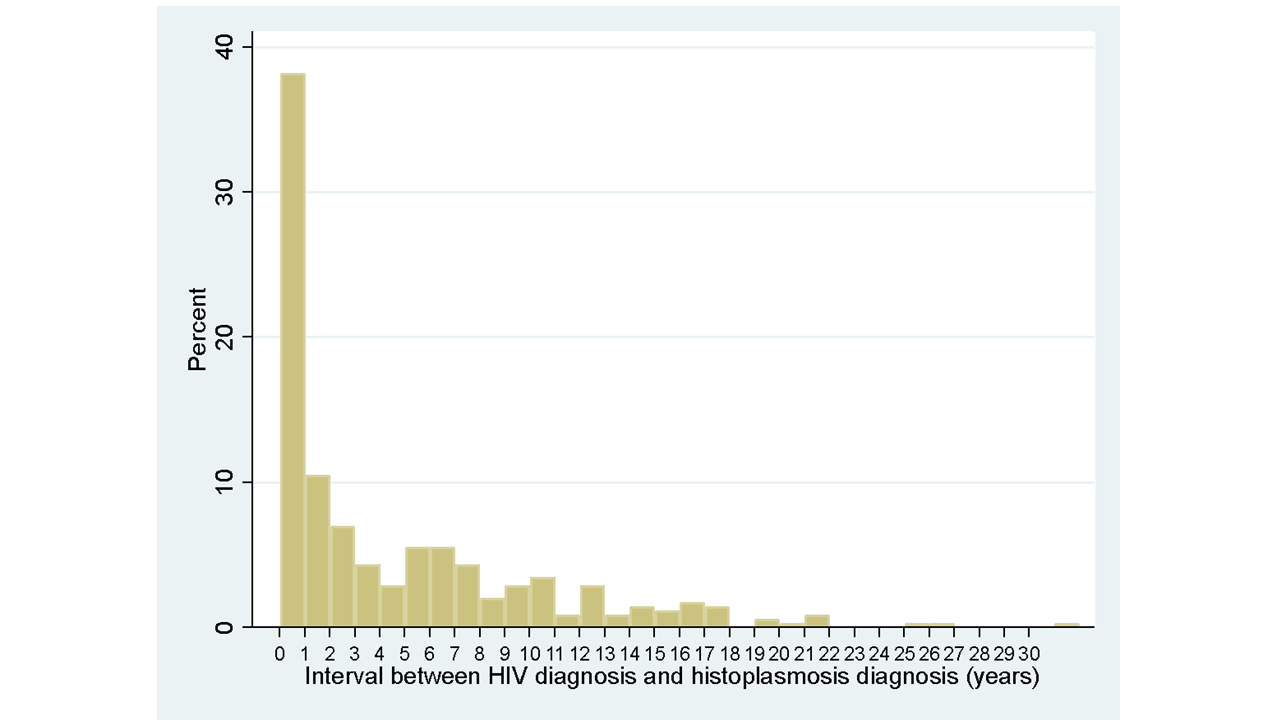

Supplement: S1 Fig — (TIF) [file pntd.0011066.s001.tif]

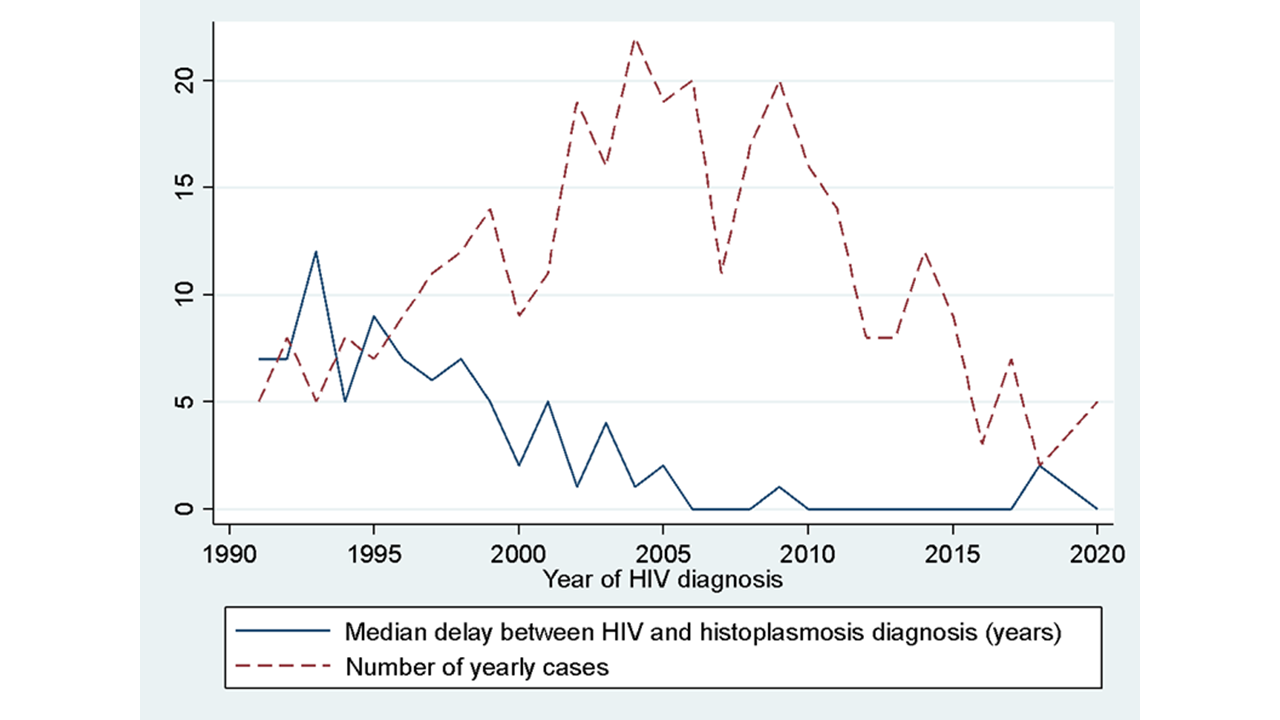

Supplement: S2 Fig — (TIF) [file pntd.0011066.s002.tif]

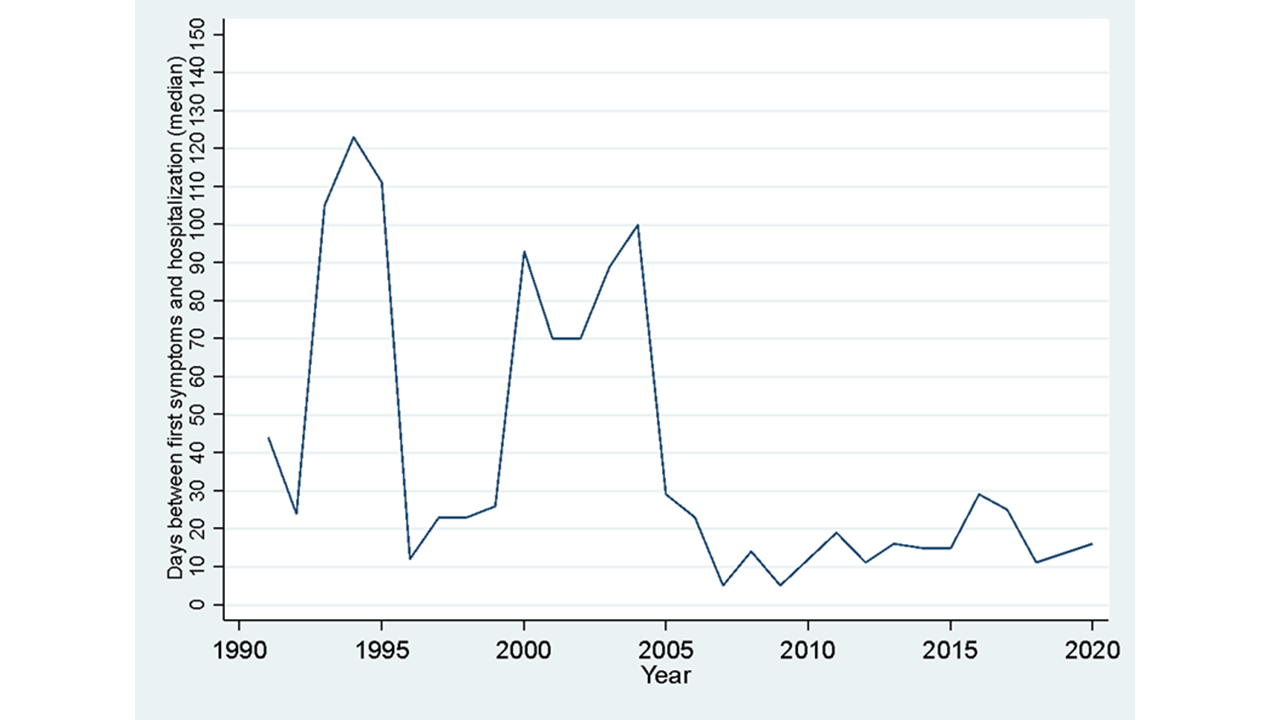

Supplement: S3 Fig — (TIF) [file pntd.0011066.s003.tif]
